# Traité Clinique du Rheumatisme Articulaire

**Published:** 1841-01-01

**Authors:** 


### Traité Clinique du Rheumatisme Articulaire, &c. Par *J. Bouillaud*. Octavo, pp. 554. Paris 1840. J. B. Baillière.

Although the previous work of M. *Bouillaud*, of which the present one may be considered as only an enlarged and amended edition, was reviewed at considerable length a few years ago in this Journal, our practical readers will probably not deem a few pages needlessly occupied if we again call their attention to the subject of Rheumatism—one of the most common, and yet one of the least thoroughly understood, ills that man's flesh is heir to. This assertion may be considered by some to be somewhat hasty and inaccurate; but if we consider for a moment the discrepancy of treatment recommended by many of the ablest physicians of the present day, not to allude to those of former times, it will be admitted that it is not so groundless as at first view may be supposed. And can we wonder that the treatment of any disease should be so undecided and fluctuating, when we call to mind that its pathology—or proximate cause, to use the language of our forefathers—is so little understood? In what other malady, except perhaps in typhus fever and neuralgia, has the treatment been more empiric and unsteady? While one physician recommends bleeding, mercury, and other antiphlogistics, another trusts chiefly to colchicum or guaiacum, and a third assures us that cinchona may be regarded as almost a specific. Surely it cannot, we might suppose, be in one and the same malady that remedies, so essentially different, are found to be equally, or nearly equally useful; unless indeed the character or type of rheumatism, like that of typhus, varies in different seasons, and is subject to the operations of the medical constitution of the air, the state of health of the patient, &c.—influences which it is unquestionably necessary to attend to in the study and treatment of all manner of fevers. But few, we believe, will be inclined to adopt this opinion; and yet it must be acknowledged that such is but a fair inference from the conflicting statements of medical men, in reference to the most successful mode of treating rheumatism. As this is a subject which has occupied much of our attention for several years past, it is possible that we may succeed in throwing a little light upon the difficulties which surround it, and that we may reconcile, at least in part, the striking differences of opinion entertained by different writers. Even although we may not get others to agree with us, the exposition of our own views will probably excite others to canvass more attentively the question at issue; and thus the cause of truth and of sound medical practice cannot fail to be essentially promoted. As it is not however our purpose to write an essay upon rheumatism, but to review briefly M. *Bouillaud's* new work, we shall only append our remarks in the way of commentary on the leading positions which he so energetically strives to enforce. These are the following:—

1. That acute, or as he prefers to call it, articular Rheumatism or Arthritis is invariably and essentially an inflammatory disease; that the inflammation has nothing specific in its nature, and that it is liable, like other forms of phlegmonous disease, to terminate in resolution, the effusion of lymph, suppuration, and ulceration.

2. That the primary seat of the inflammation is in all cases the synovial membrane of the joints, the other tissues of the joints and limbs being only secondarily affected.

3. That in almost every case of acute rheumatism, there is a *coexistent* inflammation of the lining membranes of the heart, or, in other words, pericarditis and endocarditis.

4. That acute rheumatism should be treated, as all the other active Phlegmasiæ, by vigorous depletions of blood and the use of other antiphlogistic remedies; and that, by adopting his *nouvelle formule* of bleeding *coup sur coup*, the disease may generally be cured in from one to two weeks.

Before offering any comments on these positions, it is proper to observe that M. *Bouillaud's* work does not profess to be a complete treatise on rheumatism; it is limited almost entirely to the consideration of the *acute*

form of the disease, or to what is frequently denominated, both in France and England, rheumatic fever. In this respect the work is very incomplete —as it takes only a partial view of the disease, and thus neither does justice to the subject, nor fairly enables the reader to form any conjecture in what light the author regards those very numerous cases, generally of a *chronic* nature, which are classed together, in ordinary language, under the generic term of rheumatic.

But M. *Bouillaud*, it is well known, is one of those impetuous vehement characters, who seize upon a favorite opinion, and invariably carry it *à l'outrance*. He may be truly called a man of a single idea in the practice of medicine; his mind is ever occupied with the idea of inflammation, and his treatment of almost every disease seems to consist in bleeding, bleeding, bleeding. Whoever is in the habit of perusing the French periodicals of late years, knows how largely he has contributed by his clinical reports to the diffusion of the Broussaian doctrines: and being a quick and vivacious writer, he has acquired a notable reputation among the ardent spirits of La jeune France medicale.

The disease, Rheumatism, conveys no other idea to his mind than the existence of a phlegmonous inflammation in certain tissues of the body; and its treatment therefore, according to this view, requires nothing but the usual antiphlogistic remedies. He seems to be not at all aware that there may be different *kinds*, so to speak, as well as different *degrees* of inflammatory action; and yet every reasonable writer, if he has been long conversant with clinical practice, must admit the truth of this statement. Is there not the phlegmonous, the scrofulous, the syphilitic, the rheumatic, and the gouty inflammation? The essential or *generic* nature of these various forms of the disease may be the same; probably it is; viz.—an obstructed state of the capillaries of the inflamed part, and an increased action of the larger arteries leading to it. But each form has its specific peculiarities, by which it differs from all the rest—peculiarities which depend upon the differences, whether congenital or acquired, of the constitution of the patient, or, in other words, on the state of the fluids and solids of the body.

*Phlegmonous* inflammation, as every one knows, has a marked tendency to terminate in suppuration: so likewise has the *scrofulous* inflammation, although the suppurative action which follows it has many peculiar features.

On the other hand, the tendency of *syphilitic* inflammation is to induce a peculiar ulceration, accompanied with imperfect suppuration; while the characteristic termination of the *rheumatic* inflammation is unquestionably the effusion of coagulable lymph, and that of the *gouty* inflammation is the deposition of lithic acid either around the joints or in the urinary passages. Such is the usual course of these different kinds of inflammatory action; but as there is no medical law without numerous exceptions, and as diseases are seldom uncomplicated and simple in their nature, we may *à priori* be assured that in numerous cases an existing inflammation will partake of the characters of more than one of these forms.

Thus the scrofulous inflammation sometimes approximates very closely to the phlegmonous; or it may be blended with the syphilitic, giving rise, as every one knows, to a very unmanageable form of disease. Again, the rheumatic inflammation seems sometimes to differ little from the phlegmo-

F 2

nous in the consequences which it produces, being then followed by the partial formation of purulent matter; and at other times—and this is much more frequently the case—it is co-existent with gouty disease, and then it constitutes what is usually called *rheumatic gout.*

Let us now see whether we can form any reasonable conjectures as to the cause and nature of the specific differences in these different kinds of inflammatory action.

To arrive at any accurate conclusions on this very interesting and most important subject, more attention must be given to the state of the blood, and of the secretions from it, during the progress of disease than has hitherto been done. Physicians in all countries are beginning to be more and more impressed with the necessity of admitting a *regenerated* system of Humoral Pathology; the *solidism* of the schools during the present century has been pushed to an extravagant length, and begins to share the fate of every other exclusive dogma in medicine.

Consider for a moment the condition of the blood in *genuine* rheumatic inflammation. Is it not charged with an excessive and a most abnormal quantity of fibrine or coagulable lymph? On the other hand, is not the blood, which is drawn from patients labouring under scrofulous inflammations, usually thin and watery, with an excess of serum and a deficiency of clot?* In these two kinds of inflammation, we find the *extremes* of richness and poverty in the circulating fluids; phlegmonous inflammation being, in this respect, *intermediate* between the two.

That the peculiarities of syphilitic inflammation may be attributed to the existence of a specific poison in the blood, will not be denied by any one; although we must confess that as yet we do not at all understand how this operates, or whether it produces any appreciable changes in the fluids; and it seems equally obvious that those of gouty inflammation are intimately dependent upon the presence of an unusual quantity of acid in the circulating mass; as may fairly be deduced from the chemical condition of the urine and other secretions, and the acknowledged efficacy of alkaline medicines in the cure of the disease.

As our chief object at the present moment is to endeavour to explain the phenomena of Rheumatism—*chronic* as well as *acute*—we crave our readers' especial attention to the peculiarities of the blood in the genuine rheumatic and the genuine gouty inflammations—in the one, the blood being charged with an excess of its fibrinous portion, in the other being, so to speak, impregnated or poisoned with an acid matter.

An important question is, How are these morbid states induced? A good deal of error prevails upon this subject.

It is a common opinion, that attacks of acute rheumatism are often sudden and unpreceded by any premonitory symptoms. We believe that this is quite a mistake. If the history of any case of this disease be attentively

* In estimating the proportion of the serum and the clot, we must not be guided by the simple inspection of the blood, after it has coagulated; as the clot in thin watery blood often seems to be of large size, in consequence of its retaining a quantity of the serum within it. Its firmness and resistance to the finger should always be attended to.

enquired into, it will be always found that for several weeks, or even months, previous to the seizure, the patient has been affected not only with flying pains in several of the joints, but also with headache, with vertigo and flushing of the face, not unfrequently with cough and sense of tightness about the chest, and with a general feverish state of the system, denoted by thirst and disturbed sleep, a constipated state of the bowels, and a scanty secretion of urine, which is moreover deep-coloured and of an unusually strong smell. The digestive organs do not ordinarily suffer; hence the appetite remains tolerably good, and the patient continues to live on a full diet, taking animal food once or twice daily, and drinking his beer or wine as usual. Although he feels himself not quite well, he is not *malade* enough to be confined to the house, and he therefore strives to shake off his symptoms of *malaise*, either by living more freely or by taking more than his usual exercise. Now during all this time the blood is becoming richer and more fibrinous, in consequence of the secretions being diminished; while the quantity of chyle, that is introduced into it, is as large as ever. Were the patient at this time bled, put on a low diet, and well purged, he would speedily be relieved of all his unpleasant symptoms, and spared, in all probability, the attack which is awaiting him.

But this is not done; and things go on as before, till, being exposed to wet and cold, he is seized with smart fever, and all the well-known symptoms of rheumatic fever make their appearance. The attack may seem to be sudden and unexpected; but, on further enquiry, the physician will always find that the patient has been far from well for several weeks previously. Indeed the very circumstance of the blood having acquired such an abnormal quantity of fibrine—which, be it remembered, is the very constituent of the blood which is most slowly augmented—might lead the pathologist, independently of the other phenomena of rheumatism, to the belief that the development of the genuine disease can not be effected in the course of a day or two, as may unquestionably be the case with certain of the *regular* phlegmonous inflammations, such as bronchitis, cynanche, &c. It is quite true that a patient, who was going about yesterday, may be confined to-day with severe rheumatic fever, in consequence of exposure on the preceding evening to cold damp weather;—but then the train was already laid, and it only required the spark to be applied to occasion the explosion of the disease. In many cases, an explosion never takes place; and then the patient continues to be harassed more and more with flying erratic pains, alternately better and worse with every fluctuation of the weather, while some internal organic lesion is most probably establishing itself. We are convinced that many of the most formidable changes of structure in the viscera, more especially in the organs of circulation and of the brain, are owing to this *rheumatic diathesis*—the hæmitis or inflammation of the blood of M. *Piorry* and other writers. Every physician knows how intimate is the connexion between rheumatism and organic diseases of the heart, and yet no satisfactory explanation has been given of this remarkable connexion. M. *Bouillaud* indeed talks much of the strict analogy of character in the tissues of the heart and of the joints, and he goes so far as to call the pericardium a sort of synovial capsule! But this analogy is much more in his words than in the thing itself. It has frequently occurred to us that we may solve the question, if we reflect that the blood in rheumatism is charged with an excess of fibrine, and that it must therefore have a stronger tendency, than in health, to the deposition of coagulable lymph on the inner surface and on the valves of the heart's cavities. M. *Bouillaud* asserts, and he is quite right, we believe, in his assertion, that the pericarditis and endo-carditis, which so frequently complicate the presence of acute rheumatism, are co-existent with, and not subsequent to, the affection of the joints—in other words, that there is not so much a *metastasis* as a *coincidence* of the two diseases: now this is just what we might have expected, seeing that both seem to be connected with the same condition of the circulating fluids.

........ So much for what is, in our opinion, the primary or proximate cause of genuine and uncomplicated rheumatic inflammation—an inflammation whose essential *humoral* character is an excessive predominance of fibrine in the blood. But then the genuine unadulterated disease is probably rarely met with, except in young healthy subjects, who are affected for the first or second time with acute rheumatism. Whenever the disease has recurred frequently, or has been of long duration and has already become chronic, and especially if this occurs in adult age, it is almost always complicated either with Gouty inflammation, or with some form of Neuralgia. It is this complication of different morbid states that will be found to constitute the true character of numerous cases of Chronic Rheumatism, and attention to which will explain the reason of their extreme inveteracy, and their obstinate resistance to remedial treatment. The blood either is, or has been, (and in this latter case, the ligaments of the joints, and the fasciæ, &c. have already become thickened and otherwise altered in texture), surcharged with fibrine; in addition to this state, there may be a greater or less predominance of acid in the system; and lastly there may be superadded, at the same time, some form or another of neuralgic suffering. To prove the existence of the gouty disease, we have only to test the urine with litmus paper, which will be more deeply reddened than in health; and with respect to that of neuralgia—since we know little or nothing of the state of the nerves on which it depends—we may fairly appeal to the excellent effects, in numerous cases of chronic rheumatism, of those very remedies which are known so often to relieve all painful affections of the nerves—such as sedatives, bark, arsenic, &c.

The appellation of Rheumatic Gout therefore—gouty rheumatism would be a more appropriate term—to many cases is, we believe, strictly correct; the elements of both diseases being co-existent in the constitution of the blood at the same time, and the successful treatment depending upon a due attention being paid to the removal of this complication. In other cases, neuralgic sufferings are associated with the rheumatic pains; the peculiarities of each case under this head varying according as the one or the other predominate. There is always reason to suspect this latter complication, when the pains are exceedingly sharp and darting, but remittent, when their severity is much influenced by the condition of the weather, and when temporary relief is derived from the use of stimulant sudorifics and sedatives.

Lastly, all the three morbid states—the rheumatic, the gouty, and the neuralgic—may be co-existent in the same case; and according as one or other of these states prevails, so will the case be found to exhibit more of a rheumatic, or of a gouty, or of a neuralgic character. The discriminating features of this *double* complication it is scarcely necessary to allude to, as they may be easily inferred from what we have already said.

By attending to these simple suggestions, the practitioner will often be enabled to accommodate his remedies to the peculiarities of each case; but, as we shall recur to this subject when we come to describe the treatment of rheumatism, we shall drop it for the present, and proceed to offer a few comments on the four positions, which embody the substance of M. *Bouillaud's* treatise.

The *first* of these—that acute rheumatism is simply a phlegmonous inflammation of the synovial capsules and of the other appendages of the joints, and that like other phlegmonous inflammations it may terminate in suppuration and ulceration—is not strictly correct. We have already pointed out what seem to us to be the peculiarities of rheumatic inflammation; and with respect to the latter part of the position, that it is liable to terminate in suppuration, we need scarcely remind the reader that this is only of occasional and of very rare occurrence. M. *Bouillaud* indeed has published the reports of no fewer than between thirty and forty cases of, what he considers, genuine rheumatism terminating in suppuration. But a large majority of these cannot be regarded as instances of any form of rheumatism. What other writers have described under the term purulent diathesis, M. *Bouillaud* classifies under the head of rheumatism; and by bringing all together he has succeeded in producing a famous long list of cases to maintain his position.

But such is the besetting sin of our author upon all occasions—a fondness for generalization from inaccurate principles. To do however all manner of justice to him, we shall extract a few of his cases.

*Case.*—A soldier, while recovering from what seems to have been synochus, imprudently exposed himself to cold, and was seized with a return of fever which was accompanied with violent pains in the joints, more especially in the knees. This was on the 17th of August, and he died on the 26th. On dissection, most distinct traces of endocarditis in the left side of the heart were observed; the lining membrane of the ventricle, its valves, and of the aorta was of a deep red colour, and several fibrinous and albuminous coagula were found in the cavities of the heart. The internal surface of the vena portæ, and also of the crural artery and of the internal saphæna vein, exhibited marks of inflammation. (It is stated in the report that M. *Regnault* had prognosticated during the life of the patient the existence of inflammation of the vessels in addition to acute rheumatism. Query: What are the symptoms of such a complication?) *A large quantity of yellow well-formed pus was found in the knee-joints, the synovial membranes of which were red and thickened.*

*Case.*—A young soldier, recently discharged from the Val de Grace Hospital cured of bronchitis, was re-admitted within a fortnight in consequence of a relapse. Two days afterwards the left wrist-joint became swollen and painful, and two days later the right knee, &c. was similarly affected. There had been a well marked fit of shivering before this second seizure. On the 21st (three days subsequently) the left knee and ancle-joints became painful and swollen: the constitutional symptoms seem to have been typhoid. The patient became delirious, and died on the 26th—ten days after the first appearance of articular disease.

*Dissection.*—All the affected joints were found full of a thick yellow pus; this was observed also in the sheaths of the flexor tendons of the left fore-arm, and in the interstices of the fibres of the triceps muscle of the thigh: the articular surfaces did not exhibit any abnormal appearances.

The propriety of regarding this case as one of genuine rheumatism cannot surely be admitted: the next, however, is rather more satisfactory.

*Case.*—A soldier was admitted into the Military Hospital at Strasbourg, with an attack of most severe articular rheumatism. The pains were most violent in the knees, which were outwardly red and much swollen: the shoulders and wrists were also swollen, but in a less degree. On the fifth day of the seizure, the disease concentrated itself on the knees; the pain in the other joints having ceased. Next day, all the nervous symptoms were aggravated, and on the eighth day the patient died delirious.

*Dissection.—Both knee-joints were outwardly much swollen. No sooner was an opening made into their cavities, than an immense quantity of pus flowed out: the synovial membranes were red and thickened.*

The only other case which we shall give is doubly interesting, on the one hand, from being reported with more than usual accuracy and minuteness, and, on the other, from being drawn from the practice of M. *Chomel*, between whom and M. *Bouillaud* there is a keen war of opinion as to many of the leading points in the history of acute rheumatism.

*Case.*—A middle-aged woman, of a healthy constitution, was admitted into the Hôtel Dieu, on the 3d November. For eight days, she had been suffering with severe pains in all the joints, more particularly in the shoulders and knees. Next day she was freely bled and put upon a low diet. As the pains were not relieved, she was treated with large doses of tartar-emetic, according to the Italian method. On the 11th, the pains had left the joints, but the patient was distressed with severe colic and nausea, and the pulse was extremely languid. Next day the abdominal suffering was exasperated, and was attended with profuse diarrhœa. The patient died in the course of the evening.

*Dissection.*—The joints exhibited traces of well-marked inflammation: they were all filled with a thick synovia, which was of a yellow colour, turbid, gluey, and resembling concrete oil, or rather the spermatic fluid, if that was coloured yellow. The synovial surfaces were more or less red at several points. . . . . . . . The pericardium contained two or three spoonfuls of slightly turbid fluid. Its inner surface was somewhat reddened and injected, and here and there exhibited spots of albuminous deposition.

We have already stated that most of the remaining—nearly thirty—cases, adduced by M. *Bouillaud* to prove that acute rheumatism may terminate in suppuration of the affected joints, are far from being satisfactory: most of them, unquestionably, cannot be regarded as examples of *genuine* rheumatism. For example, six of them occurred during the puerperal state, and on dissection in some of these all the traces of metritis, accompanied with suppuration of the uterine veins, were found. Surely no pathologist, except our author, would regard such cases as belonging to *genuine* rheumatism.

In another case which occurred in a man who died with hydrothorax, and who for a week or two before death had been affected with severe pain in the right knee which was much swollen, a vast abscess was found on dissection among the muscles of the thigh, and a quantity of puriform matter in the capsule of the knee-joint.

Such a case can only be viewed as one of local arthritic disease, and has scarcely any thing in common with the essential characters of acute rheumatism. The same remark is applicable to several of the other cases reported at length by M. *Bouillaud,* who, with his usual zeal to establish a favourite doctrine, has admitted not a few instances of disease without much regard to pathological accuracy.

It is well known that in some cachectic states of the system—as, for example, after wounds and injuries, as well as during the puerperal state, and during phlebitis, and erysipelas—there is a peculiar tendency to an unhealthy inflammation of the joints taking place, and to the formation of purulent deposits in their cavities, and also in some of the parenchymatous viscera of the body. Our author indeed seems to be quite aware of this objection; but, with his characteristic skill in controversial fencing, he tries to evade its force, by telling us that rheumatic inflammation sometimes affects the internal surface of the bloodvessels, and that, in the cases we have alluded to, there was a rheumatic phlebitis or arteritis present. We may admire the ingenuity of the defence, but we cannot admit its force.

Let it not, however, be imagined that we withhold all praise from M. *Bouillaud;* we cheerfully acknowledge that he has contributed to throw some light on the pathology, still very obscure, of rheumatism, by bringing together the 37 cases, which he has reported; and although most of them must unquestionably be put *hors de combat,* we admit that several of them may fairly be regarded as examples of rheumatism terminating in partial suppuration of the affected joints.

And can we wonder at such an occurrence occasionally? Assuredly not; for, although suppuration is a rare sequence of rheumatic disease, cases do occur, where, from the influence of particular states of the constitution, or from the protracted duration of the disease, or from the operation of remedial agents that may have been used, or, lastly, from the co-existence of other morbid states in the system at the time,* the inflammation partakes much of the character of ordinary phlegmonous inflammation. We have already distinctly stated that, in a great number of cases called rheumatic, the disease is not simple, uncomplicated rheumatism, but a complex morbid state, the existent inflammatory action varying in its characters, and being associated, or not, with neuralgia at the same time. On the whole, therefore, we are bound to assent to the hitherto generally received

---

* M. *Bouillaud* gives it as his opinion, that the chances of a suppurative termination are proportional to the severity of the preceding rheumatic inflammation —a doctrine certainly not at all warranted by experience. Perhaps, indeed, the very reverse of this doctrine would be nearer the truth.

doctrine, that suppuration and ulceration are very rare, although certainly occasional, consequences of acute rheumatism. They have been usually observed in cases of very long duration, and when the disease has for some time fixed itself upon one joint in particular.

With respect to the *second* position of M. *Bouillaud*, that in acute rheumatism the synovial membranes of the joints are the parts primarily and essentially affected, we shall not detain the reader long. Unquestionably in a large number of cases this is strictly true. But that the aponeuroses or fasciæ of the muscles also, if not the muscular tissue itself, the bursæ mucosæ and the sheaths of the tendons are very often from the first the seat of the disease, cannot be denied. Some excellent practical writers have distinguished two forms of acute rheumatism—the *fibrous*, in which the aponeuroses, tendons, and muscles are chiefly affected, and the *synovial*, in which the lining membranes of the joints are primarily and essentially involved; and they have shewn that these two forms of the disease require certain specialties of treatment, to which we shall presently allude.

The *third* position of our author, that in the majority of cases of acute rheumatism there is a *coincidence* of pericarditis or endocarditis, or of both, is one of great interest, and explained and illustrated by him with much ability and zeal.

" Of 114 cases of acute articular rheumatism," says he, " of which we have kept a most minute record, in 74 the symptoms were severe, and in 40, they were much milder. Now of the 74, the coincidence of endocarditis or of endo-pericarditis was ascertained in 64 beyond doubt; whereas in the 40 cases there was not one in which this coincidence could be detected."* M. *Bouillaud* will not allow that in any case a genuine *metastasis* or *translation* of the inflammation from the joints to the heart ever occurs. Here again we have another instance of his fondness for extreme opinions. Because, in a number of cases, the affection of the joints and that of the heart are coincident or co-existent, he insists that the disease is never suddenly transferred from the former to the latter; although he afterwards acknowledges that " sometimes the endocarditis and the pericarditis appear subsequently to the affection of the joints, and, at the period of their development, this (the articular affection,) becomes very sensibly diminished;" adding, " but this does not authorize us to say that there is a veritable *metastasis*, as it rather seems in such a case that the affection of the heart acts, so to speak, like a *blister!*"

The following admirable remarks by M. *Andral*, taken from his notes on *Laennec's* immortal work, while they do all manner of justice to the merits of our author, stop short of his *exclusivism*.

" The researches of M. *Bouillaud* have shewn that there is a much more frequent *coincidence* between rheumatism and certain affections of the heart, than had been suspected before. In the present day we can no longer doubt that, in a great number of cases of acute articular rheumatism, the internal membrane of the heart has a singular tendency to become inflamed........ For my own

---

* He adds that, in more than one-half of 300 cases of organic disease of the heart, examined by him, the patients had been affected at some previous period with rheumatism.

part, I no longer question the important influence of acute articular rheumatism in the production of acute diseases of the heart. On the one hand, I am convinced, by attentive investigation, that a considerable (assez grand) number of persons affected with various lesions of the heart have at some antecedent period suffered from acute rheumatism, and that it was from, or shortly after, this date that they began to experience some uneasy feelings in the cardiac region: and, on the other hand, having in a number of rheumatic patients watched the state of the heart day after day, I have in some measure heard the affection of this organ rise under my ear. At first, in some cases during the existence of, and in other cases after the cessation of, the articular pains, we perceive a blowing sound, which, feeble at its commencement, becomes daily more and more distinct. At this period, there is generally neither pain, nor palpitation, nor dyspnœa; subsequently, these two last symptoms make their appearance, and denote in most cases an incipient hypertrophe of the heart, which is the result of the endocarditis—the primary lesion induced by the rheumatism. I have seen other cases where, several years after an attack of acute rheumatism, no other morbid symptom of the heart, except a blowing sound, could be detected. In such a case we must admit that there is a contraction of one of the orifices of the heart, unaccompanied (which is very rare) with any thickening of its parietes or any enlargement of its cavities."*

It is unnecessary to dwell further upon the extreme importance of early attention to the occurrence—whether this be alarmingly rapid, or insidiously slow—of any thoracic distress during, or for some time after, an attack of acute rheumatism.

If, under such circumstances, any degree of dyspnœa comes on, or if a feverish restlessness, increased at night, continues after the cessation of the articular pains, there is reason to suspect that some mischief is going on in the heart or lungs, or in both of these organs. Should prompt relief not be given, and especially if the error be committed, unfortunately not an unfrequent one, of attributing the symptoms to debility and spasm,—in consequence, perhaps, of the debility of the pulse and the occasional intermission of the dyspnœa—and treating them as such, the chances are that the patient will be lost.†

We have left ourselves but little space to comment on the *last*, and the most important position—that which respects the treatment of the disease.

The plan, which M. *Bouillaud* usually follows, is to bleed his patients

---

* In the Medico-Chirurgical Review for October 1827, will be found some remarks which, while they anticipate, strikingly confirm these observations of M. *Andral*. In one sentence it is stated:—"For many years past we have paid considerable attention to diseases of the heart; and, on minute enquiry, we have found that in the majority of cases there had been one or more attacks of acute or sub-acute rheumatism primarily. *There may be no direct metastasis at the time the acute rheumatism occurs—but the disease of the heart often steals on afterwards, without any other ostensible cause than the rheumatic diathesis.*"—p. 346.

† In the Foreign Periscope of our present number will be found the report of a most interesting case, from the private practice of M. *Bouillaud*, which illustrates in a very striking manner the danger of inaccurate diagnosis, and at the same time the success which often attends the adoption of vigorous antiphlogistic treatment.

twice on the day that he first sees them, and then once every succeeding day, independently of leeches and cupping, until the active symptoms are subdued. The average quantity of blood, drawn in severe cases, may be stated at from five to six pounds in the course of three or four days; and in less severe cases at about four pounds and a half in between two and three days. In some unusually severe cases, as much as eight, nine, or even ten pounds of blood have been drawn, before the resolution of the symptoms was fairly established; " but," adds M. *Bouillaud*, " I have lost none of those patients in whom the disease had attained *this extreme gravity*, by adopting *this extreme remedy*." The auxiliary or adjuvant means, which he recommends, are poultices, blisters, and afterwards moderate compression with bandages and pledgets smeared with mercurial ointment, or wetted with an astringent wash. " Opium, administered either inwardly or endermically, low diet, and diluent drinks, complete the list of our principal adjuvant remedies."

The effect of the treatment now recommended is, we are told, "to reduce the mortality of the disease to *Zero;* to prevent its lapsing into a chronic state, and also the insidious development of cardiac disorder; and, lastly, to abridge its average duration from six or eight to one or two weeks."

As to the treatment of the *chronic* forms of rheumatism, recommended by M. *Bouillaud*, it consists in general and local bleeding, although to a less extent than in the acute disease, and in the use of blisters, moxas, opiate preparations, internally as well as externally administered, vapour or sulphurous baths, and compression of the joints.

It is scarcely necessary for us to express our opinion as to the great defects of our author's *treatment* of rheumatism, whether acute or chronic. It will be observed that some of the most potent remedies are not even mentioned by name—as mercury, iodine, colchicum, and guaiacum. The extravagance of his bloodletting practice must at once be denounced as decidedly most injurious. It is rarely necessary to bleed from the arm oftener than twice or thrice at the utmost, provided we resort to the use, at the same time, of some of these internal remedies. From the almost specific effects of *mercury* in arresting inflammatory action, and in acting somehow on the constitution of the blood itself—rendering it less viscid and counteracting the tendency to fibrinous depositions—it forms one of the most potent of all anti-rheumatic remedies. The great objection to its use is the exceeding annoyance of the salivation that is so apt to be induced; but, in spite of this inconvenience, we believe that it should seldom be omitted in the treatment of acute rheumatism.

Iodine also, more especially its salt, the hydriodate of potash, is another excellent remedy. There is reason to believe that its action on the system is similar in many respects to that of mercury, although it is decidedly inferior to it in efficacy.

The Guaiacum mixture of the Pharmacopœia, used freely after bloodletting and purging, has been tried very largely by Dr. *Seymour* of St. George's, and found by him of almost unerring success in the severest forms of acute *fibrous* rheumatism: it acts as a general evacuant, provoking perspiration, purging, and a copious flow of the urine. The colchicum, another powerful remedy, is better suited for cases of the *synovial* form of the disease. Cinchona is always a precarious, and often a most pernicious, medicine in acute rheumatism; in enfeebled constitutions however, it, and especially quinine, may be administered along with mercury or guaiacum, to enable the system to bear the effects of these remedies. Opium cannot be trusted to alone, or be safely used at the commencement of the disease, as recommended by some physicians; although it is certainly the most powerfully efficient of all remedies, when the violence of the inflammatory action is arrested, and when the pains have become rather of a spasmodic than of a phlogistic nature: under such circumstances, a grain of opium every four or six hours will often act as a charm.

In adapting our treatment to each case, we should always keep in mind the morbid condition of the blood that is present in all. While it is surcharged with fibrine, bleeding, with the usual antiphlogistic remedies, and the use of mercury, iodine, and such a general evacuant as guaiacum, constitute by far the most efficient and certainly successful practice.

The treatment of the numerous ailments comprehended under the term of Chronic Rheumatism, is accompanied with far greater difficulty; still, much of this may be got rid of by attention to the remarks which we have already made as to the various morbid states which complicate, or even entirely replace, the original disease. The reason that so many cases of what is called Chronic Rheumatism are so intractable is, that there are several maladies coexisting at one and the same time; but we need not now recur to this subject. Suffice it to say, that whenever we have reason to suspect, in chronic rheumatism, that the blood is still buffy, the detraction of a moderate quantity will unquestionably facilitate the cure. In protracted cases, it is useful to make an *explorative* bleeding to a small amount, for the purpose of ascertaining if this condition of the blood be present; for if it is, the most successful internal treatment will be found to consist in the use of mercury —the oxymuriate or bichloride may often be used with admirable effects— or of iodine, or of guaiacum. If, on the other hand, the blood be not at all buffy, the probability is, that the so-called rheumatic pains either are connected with a gouty state of the system, or are chiefly of a neuralgic character.

In the first case, a course of alkaline remedies and of colchicum, coupled with an occasional purgative and abstinence from all fermented and vinous liquors, should constitute the basis of the treatment; and, in the latter case, the use of sedatives and tonics, more especially of bark, arsenic and such like remedies, not omitting the exhibition of alterative aperients,* promises by far the best chances of relief. To subdue local pains, blisters and sinapisms are usually much more effectual than the embrocations which are commonly used.

---

* The addition of small doses of Croton oil to aperients seems often to exert a very useful effect in protracted cases of neuralgic suffering. The exhibition of occasional emetics also is not unfrequently an excellent adjuvant: a full dose of an anodyne given after the operation of free vomiting will often soothe the most excruciating pain, when the anodyne alone would have failed.

